# Antifatigue effects and antioxidant activity in polysaccharide fractions from Chinese *yam bulbils*

**DOI:** 10.1002/fsn3.3836

**Published:** 2023-11-21

**Authors:** Hai‐Xu Zhou, Xiao Zhang, Ren‐gui Huang, Tong‐chao Su

**Affiliations:** ^1^ Henan Institute of Science and Technology Xinxiang China; ^2^ Chongqing SIIE Product Quality Testing Co., Ltd. Chongqing China

**Keywords:** antifatigue, antioxidant, Chinese *yam bulbils*, exhaustive swimming tests, polysaccharide

## Abstract

Polysaccharides are the principal component in Chinese *yam* (*Dioscorea opposita* Thunb.) *bulbils*. The properties and antifatigue of polysaccharides from *yam bulbils* (PYB) were identified and compared. Their molecular weights (PYB‐1 and PYB‐2) were approximately 145 and 11 kDa, respectively, with active β‐configurations. Meanwhile, the antifatigue activities of PYBs were tested in mice via exhaustive swimming tests (EST). The EST results indicated that PYB‐1 and PYB‐2 significantly prolonged swimming time in mice (*p* < .05). Associated with this increase was a rise in hepatic glycogen content and antioxidant enzyme (superoxide dismutase (SOD), glutathione peroxidase (GSH‐Px)) activity, along with a decline in blood urea nitrogen, lactic acid, and malondialdehyde levels. The results showed that molecular weight might contribute to the antifatigue effects of PYBs. Additionally, antioxidant tests showed that PYB‐1 had stronger free‐radical scavenging activity than PYB‐2. Taken together, the findings indicated that PYBs exhibited effective antifatigue and antioxidant activities providing additional evidence supporting the use of PYBs as functional food ingredients for relieving fatigue.

## Graphical abstract

Two polysaccharides (PYB‐1 and PYB‐2) were extracted from Chinese *yam bulbils* with antifatigue effects related to the molecular weight. This finding will enhance the economic value of Chinese *yam bulbils* and benefit the industry.

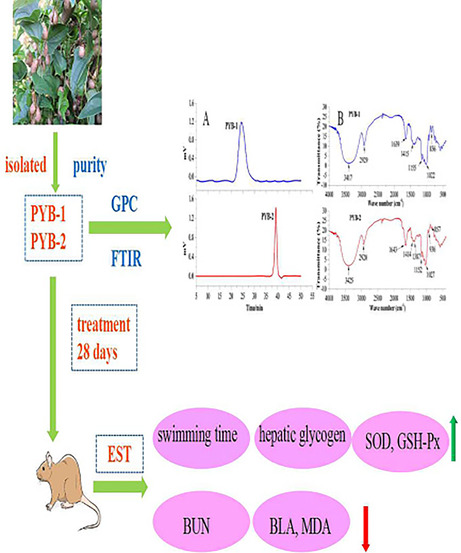

## 1 INTRODUCTION

Chinese *yam bulbils*, commonly known as “shanyao dan,” are the axillary buds of *Dioscorea opposita* Thunb. They are mostly cultivated in Henan, Anhui, Hubei, and Jiangxi Provinces of China; in Henan alone, the 2021 yield reached 4500–7500 kg/hm^2^ (Liu, Gou, et al., 2022). Chinese *yam bulbils* are cheap and rich in nutrients, containing polysaccharides (including mucus‐like substances and glycoproteins), proteins, polyphenols, flavonoids, and other beneficial compounds (Wang et al., 2020). These components contribute to the potential health benefits of Chinese *yam bulbils*, including their antioxidant and hypoglycemic effects. However, these bulbils are primarily used for seeding, reproduction, and cultivation, with only a small proportion applied to generating healthy food. Additionally, research on Chinese *yam bulbils* focuses mainly on the yield of crude polysaccharides, little research has been performed about the specific compositions and their antifatigue activities.

Fatigue is a complex physiological and biochemical process that results in decreased activity levels, reduced vitality, and compromised general well‐being (Cui et al., 2021; Hu et al., 2020; Li et al., 2020; Zhu, Yang, et al., 2021). Fatigue is related to aging and various diseases such as multiple sclerosis, cancer, depression, and Parkinson's disease (Nakagawasai et al., 2021; Wang et al., 2021). Typically presenting as weakness, lethargy, and exhaustion, fatigue affects work productivity and resistance to illness (Cai et al., 2021; Chi et al., 2015; Hsu et al., 2018; Hu et al., 2020; Tung et al., 2019). Given these undesirable outcomes, a food that can effectively combat fatigue may have important health benefits. Pharmaceutical drugs are effective for treating fatigue but may cause side effects. For example, vigor‐enhancing medicines often include ingredients such as stimulants that have negative physiological consequences (Yang et al., 2019).

Research in recent decades has found that natural compounds tend to reduce fatigue with fewer side effects (Zhu, Yi, et al., 2021). In particular, polysaccharides come from plants such as *Lepidium meyenii* Walp. (maca) (Li et al., 2017), ginseng (Jiao et al., 2021), *Stigma maydis* (corn silk) (Zhao et al., 2017), *Trichilia catigua* (catuaba), and *Ribes stenocarpum* Maxim (Qiao et al., 2022) have all been shown to exhibit antifatigue activity. Some studies have indicated that polysaccharides exert antifatigue activities, such as scavenging free radicals, inhibiting lipid peroxidation, and inhibiting linoleic acid oxidation (Cai et al., 2021). PYB, polysaccharides in Chinese *yam bulbils*, are the principal water‐soluble compound. Research using ultrasound‐assisted extraction (UAE) has confirmed that PYB exhibits antioxidant activity and hypoglycemic effects (Zhou, Huang, et al., 2021), but no data are available on potential antifatigue activity. Identifying this health benefit in PYB and clarifying the underlying mechanisms would benefit efforts to address fatigue without unwanted side effects. Furthermore, such a development will enhance the economic value of Chinese *yam bulbils* and benefit the industry.

**SCHEME 1:**
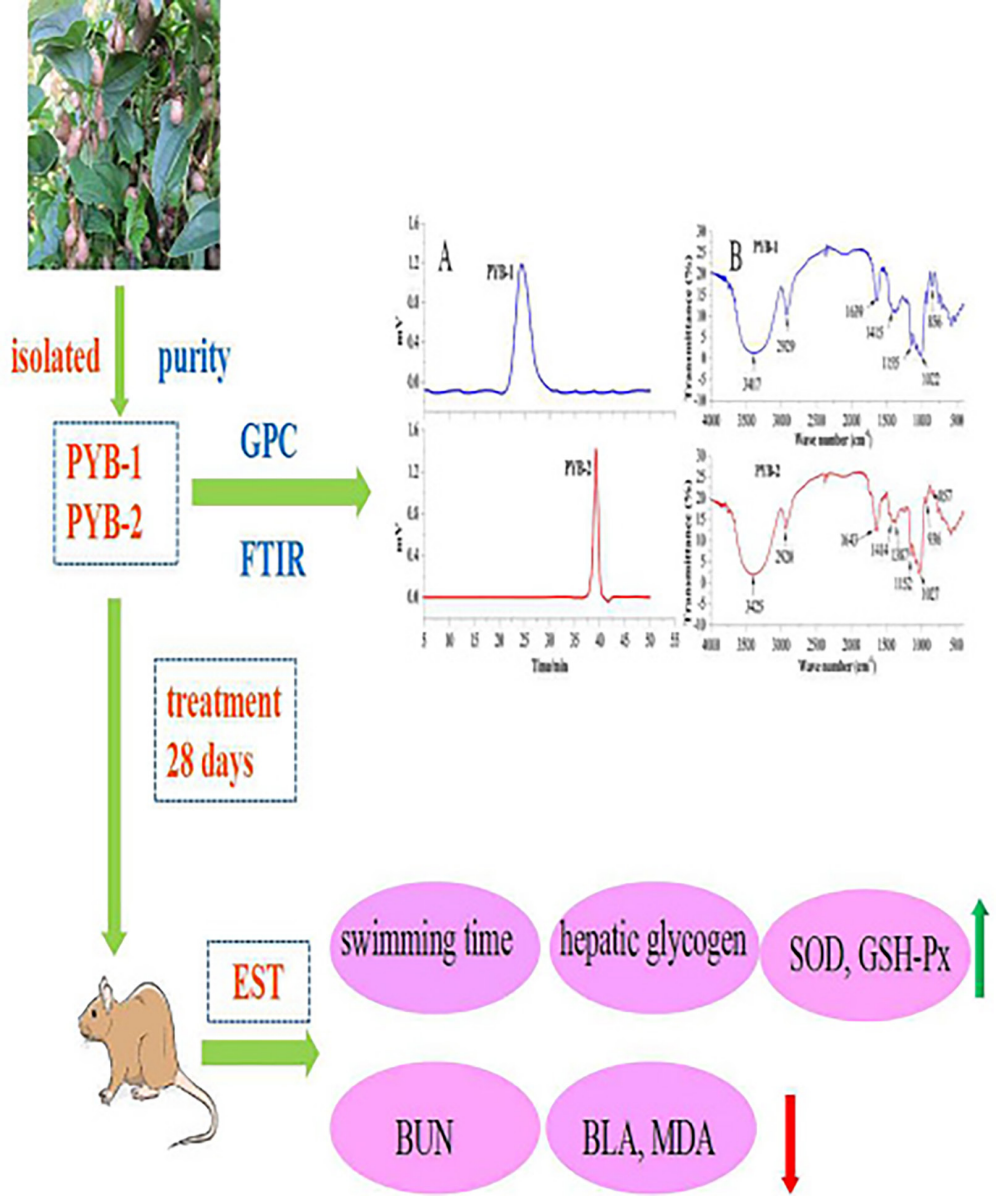
Schematic representation of the experimental design for evaluating PYB (polysaccharides from Chinese *yam bulbils*) antifatigue activity in mice subjected to exhaustive swimming tests.

It is well known that the biological activities of polysaccharides are closely related to their structural properties, such as molecular weight, monosaccharide composition, and glycosidic linkage (Cai et al., 2021; Jiang et al., 2023). Therefore, in this study, PYB were predicted that they exert antifatigue effects which were related to their molecular weight or monosaccharide composition. To illustrate the hypothesis, a series of works were done. Firstly, two polysaccharides from Chinese *yam bulbils* were extracted and purified. Then, PYB structure was characterized by molecular weight, monosaccharide composition, and other properties. Next, the mice were treated with purified PYB and subjected to exhaustive swimming tests (EST); through these experiments, the fatigue‐related biochemical parameters were measured to verify PYB antifatigue activity (Scheme 1). The findings will provide application support for using PYB in nutrition science. Furthermore, to the best of our knowledge, this is the first study to characterize PYB structure and compare the antifatigue effects of two polysaccharides.

## 2 MATERIALS AND METHODS

### 2.1 Materials

*Yam bulbils* were purchased from Jianguo Pharmaceutical Co. Ltd. (Jiaozuo, Henan, China). Commercially available large plant Rhodiola capsules (LPRC), employed as the positive control group (PG), were purchased from Kanion Pharmaceutical Co. Ltd. (Jiangsu, China). Assay kits for evaluating biological indicators (blood lactic acid, BLA; malondialdehyde, MDA; blood urea nitrogen, BUN; hepatic glycogen; superoxide dismutase, SOD; and glutathione peroxidase, GSH‐Px) were purchased from Nanjing Jiancheng Biotechnology Institute (Nanjing, Jiangsu, China). Standard sugars (rhamnose, Rha; glucose, Glu; mannose, Man; galactose, Gal; arabinose, Ara; and xylose, Xyl), were purchased from Sigma‐Aldrich (St Louis, MO, USA). Other reagents were of analytical grade.

### 2.2 Isolation and analysis of PYB

Polysaccharides were isolated from Chinese *yam bulbils* and extracted using UAE as previously reported (Li et al., 2017), with some modifications. The equipment used was an ultrasonic extraction device equipped with a time controller (JY92‐II, Ningbo Scientz Biotechnology Co., Ltd., Ningbo, Zhejiang, China). Freeze‐dried and powdered *yam bulbils* were soaked in 25°C distilled water at a ratio of 1:18 (m/v) for 24 h. An extract was prepared using UAE (760 W) at 60°C for 23 min. The supernatant was centrifuged (5000 × *g*, 20 min, 25°C), collected, and concentrated to 20% of original volume using rotary evaporation (RE‐52AA, Shanghai Arong Biochemical Co., Ltd., Shanghai, China) at 50°C. The concentrate was precipitated with anhydrous ethanol (three‐fold volume) at 4°C overnight, and then freeze‐dried for 24 h to obtain crude PYB.

### 2.3 Purification and chemical composition analysis of PYB

Proteins in crude PYB were removed as described by Li et al. (2017) using the Sevage method and papain enzyme. The phenol–sulfuric acid method was adopted to measure PYB concentration after deproteinization, with Glu as a standard (Zhao et al., 2021; Zhou, Cao, et al., 2021; Zhou, Huang, et al., 2021). Crude PYB powder was mixed with distilled water (1:20, w/v) and repeatedly subjected to column chromatography on DEAE‐52 cellulose (PH9079, Phygene Life Sciences Company, Fuzhou, Fujian, China). The mixture was eluted with distilled water and different NaCl concentrations (0.1–1.0 mol/L) at a rate of 0.3 mL/min and then subjected to an automatic fraction collector (15 min per tube). Fractions were further purified via the Sephadex G‐100 gel (9050‐94‐6, Shanghai Zheyan Biotech Co. Ltd., Shanghai, China) and distilled water at a rate of 0.5 mL/min (10 min per tube). Column fractions were detected via the phenol–sulfuric acid method, which combined samples with relatively higher polysaccharide content. Next, fractions were collected, concentrated, and freeze‐dried. Before further analysis, PYB purity and characteristics were determined using ultraviolet–visible spectrophotometry (UV1800, Shimadzu, Kyoto, Japan), high‐performance liquid chromatography (HPLC, LC‐20A, Shimadzu), Fourier‐transform infrared (FTIR) spectrometry (IRTracer‐100, Shimadzu), gel permeation chromatography (GPC, EC2000GPC, Elite, Dalian, China), and scanning electron microscopy (SEM, Quanta FEG250, FEI, Hillsboro, OR, USA).

#### 2.3.1 Monosaccharide composition of PYB

The main monosaccharide components of PYB were determined using HPLC with an ultraviolet detector at 250 nm. Parameters were set as previously described (Xu et al., 2016): Column Crack ODS‐2, 4.6 × 250 mm; 5 μm particle size (Waters Corp., Milford, MA, USA). The isocratic elution solvent was 0.02 M phosphate‐buffered saline: acetonitrile (83:17, v/v); the pH of phosphate‐buffered saline was 6.7. Solvent flow was 1 mL/min, injection volume was 0.02 mL, and column temperature was 30°C. The correlation between peak height and concentration of an external sugar standard (Sigma, 98%) was used to determine PYB composition.

#### 2.3.2 Molecular weight of PYB

Following published methods (Ebrahimi et al., 2021), GPC was used to determine PYB molecular weights (Mw). Samples (2 mg/mL, 20 μL) were injected into the GPC instrument, with a mobile phase of double‐distilled water (1 mL/min). To obtain Mw, a calibration curve was calculated between retention time of standards and the logarithm of their Mw.

#### 2.3.3 FTIR spectroscopy analysis

The chemical bonds and functional groups of PYB were identified using FTIR spectrometry. First, PYB was mixed with KBr at a ratio of 1:100 (mg/mg) and then scanned at a range of 4000–400 cm^−1^. KBr was used to minimize background interference.

#### 2.3.4 Morphological features of PYB

Morphology was observed under scanning electron microscopy (Quanta FEG250, FEI, Hillsboro, OR, USA). Measurements were conducted after freeze‐drying and gold‐spraying, and images were collected at an accelerating potential of 20 kV. Micrographs were obtained at ×600 magnification.

### 2.4 In vitro antioxidant activity of PYB

#### 2.4.1 2,2 Diphenyl‐1‐picrylhydrazyl scavenging ability

Following published methods (Mousavian et al., 2022) with some modifications, 2,2 diphenyl‐1‐picrylhydrazyl (DPPH) was used to measure PYB free‐radical scavenging capacity. First, PYB was dissolved and diluted with distilled water to different concentrations (0.2–1.0 mg/mL). Next, a 2‐mL sample was mixed with 2‐mL DPPH–ethanol solution (0.15 M) before incubation in the dark at 37°C for 30 min. Absorbance was then measured at 517 nm. Vitamin C (Vc) was selected as the positive control. DPPH scavenging rate was calculated as follows:

(1) $$\text{DPPH scavenging rate}\;\left(\%\right)=\frac{A_{\text{control}}-\left(A_{\text{sample}}-A_{\text{blank}}\right)}{A_{\text{control}}}\times 100$$

where *A*_control_ is control (distilled water + DPPH solution) absorbance, *A*_sample_ is sample (sample + DPPH) absorbance, and *A*_blank_ is blank (sample + ethanol) absorbance.

#### 2.4.2 Superoxide anion scavenging ability

Superoxide anion ($O_{2}^{-\cdot }$) scavenging activity of PYB was measured following published methods (Wang et al., 2022). First, 0.2 mL of PYB solution at different concentrations (0.2–1.0 mg/mL) was added to 5.7 mL of Tris–HCl buffer solution (0.05 M, pH 8.2), then incubated at 25°C for 25 min. Next, 0.1 mL of phloroglucinol (6 mM) was added to the mixture and reacted at 25°C for 5 min. Absorbance was measured at 320 nm, with Vc as the positive control. $O_{2}^{-\cdot }$ radical scavenging rate was calculated as follows:

(2) $$O_{2}^{-\cdot }\;\text{scavenging rate}\;\left(\%\right)=\frac{A_{0}-\left(A_{1}-A_{2}\right)}{A_{0}}\times 100$$

where *A*_1_ is the absorbance of mixture with phloroglucinol and sample solution, *A*_2_ is the absorbance of mixture with water and sample solution, and *A*_0_ is the absorbance of mixture with water and phloroglucinol.

#### 2.4.3 Hydroxyl scavenging ability

Hydroxyl (·OH) scavenging activity of PYB was measured following Qiao et al. (2022), with some modifications. First, PYB was diluted with distilled water to different concentrations (0.2–2.0 mg/mL). Next, 1 mL of 1,10‐phenanthroline (1.5 mM), 1 mL of FeSO_4_ (1.5 mM), and 1.0 mL of sample solution were sequentially added to phosphate buffer solution (PBS, 2 mL, 0.2 M, pH 7.4). Finally, 1‐mL H_2_O_2_ (0.01%, m/m) was added, and the mixture was incubated at 37°C for 1 h. Absorbance was measured at 320 nm, with Vc as the positive control. ·OH scavenging rate was calculated as follows:

(3) $$\cdot \mathrm{OH}\;\text{scavenging rate}\;\left(\%\right)=\frac{A_{\text{sample}}-A_{1}}{A_{0}-A_{1}}\times 100$$

where *A*_sample_ is the absorbance with sample solution and H_2_O_2_, *A*_1_ is the absorbance with distilled water and H_2_O_2_, and *A*_0_ is the absorbance of distilled water.

### 2.5 Experimental animals

Mice (20–22 g) were obtained from Hunan Silaike Jingda Experimental Animal Co. Ltd. (Changsha, Hunan, China). Subjects were housed in individually ventilated cages (five mice each) in a specific pathogen‐free environment at 20–24°C with a relative humidity of 45 ± 75% and 12/12‐h light–dark cycle. Mice were fed a chow diet and distilled water ad libitum. All experiments followed the National Institutes of Health (NIH) Guide for the Care and Use of Laboratory Animals and were approved by the Committee of Experimental Animal Welfare and Ethics of Henan Institute of Science and Technology, at the College of Animal Science and Veterinary Medicine (approved on 16 March, 2021; approval number PZASVM2103160016).

### 2.6 Exhaustive swim tests

Following a 7‐day acclimatization, mice were randomly divided into eight groups of 18 mice each: control (CG), given distilled water only; low‐dose PYB (LG), treated with 50 mg/kg body weight (BW) of PYB; medium‐dose PYB (MG), treated with 100 mg/kg BW of PYB; high‐dose PYB (HG), treated with 200 mg/kg BW of PYB; and LPRC as the PG, treated with 100 mg/kg BW of LPRC. Treatments were administered by gavage at 09:00 h daily for 28 days, and the mice were weighed every 3 days.

At 1 h after the final administration, mice were subjected to EST following the previously reported protocol (Wang et al., 2020), with some modifications. Lead weights, equivalent to 5% of subject body weight, were attached to the tail before mice were placed in a swimming pool (43 × 30 × 24 cm) filled with water at 24 ± 1°C.

Once subjects were floating, they were forced to keep swimming via gentle churning of the water surface with a glass rod. Exhaustion time was recorded when they failed to surface after 5 s (Chi et al., 2015); corresponding biochemical parameters were subsequently determined.

### 2.7 Analysis of biochemical indices

After EST, mice were immediately removed from the water, dried with a paper towel, and rested in their home cages for 20 min before being euthanized, following published methods (Thibodeau et al., 2004). Eyes were removed and whole blood samples were collected from the orbital sinuses (Moody et al., 2015). Serum samples were centrifuged for 15 min at 5000 × *g* and 4°C, then stored at 4°C until further analysis. Livers, spleens, and lungs were resected and weighed for determining organ indices with the equation Oi (%) = *W*_0_/*W* × 100, where *W*_0_ is the organ weight and *W* is the mouse weight. Livers were stored at −70°C until needed for analysis of glycogen content. Serum biochemical indicators (BUN, BLA, MDA), hepatic glycogen content, and liver oxidative stress indices (SOD and GSH‐Px) were measured using relevant commercial kits.

### 2.8 Statistical analysis

All experiments were conducted at least three times. Data are expressed as the mean ± SD. Between‐group differences were determined via one‐way ANOVA in SPSS version 17.0 (SPSS, Inc., Chicago, IL, USA). Significance was set at *p* < .05.

## 3 RESULTS

### 3.1 Isolation and purification of PYB

**FIGURE 1:**
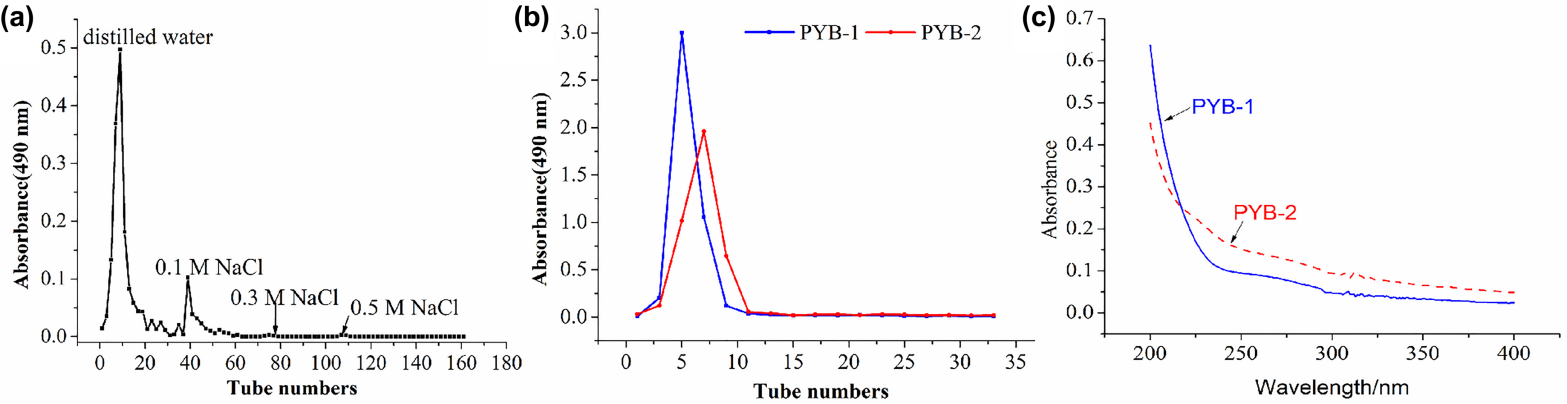
DEAE‐52 cellulose (a), Sephadex G‐100 (b) column chromatography, and ultraviolet spectrophotometry (c) of polysaccharides from *yam bulbils*.

After UAE, crude PYB yield was approximately 61.94% (w/w). Because proteins were not detected in crude PYB after initial deproteinization using the Sevage method and papain, then, further purified the extracts using column chromatography (Figure 1a,b). The main fractions eluted with distilled water (PYB‐1) and 0.1 M NaCl (PYB‐2) were further purified using gel chromatography (Figure 1b). Proteins were not detected in the PYB‐1 and PYB‐2 fractions, according to their ultraviolet spectra (Figure 1c), but one symmetrical peak was observed, demonstrating high purity. Total carbohydrate content of PYB‐1 and PYB‐2 was 89.18 ± 1.83% and 63.55 ± 1.21%, respectively.

### 3.2 Monosaccharide composition and structural characteristics of PYB

**FIGURE 2:**
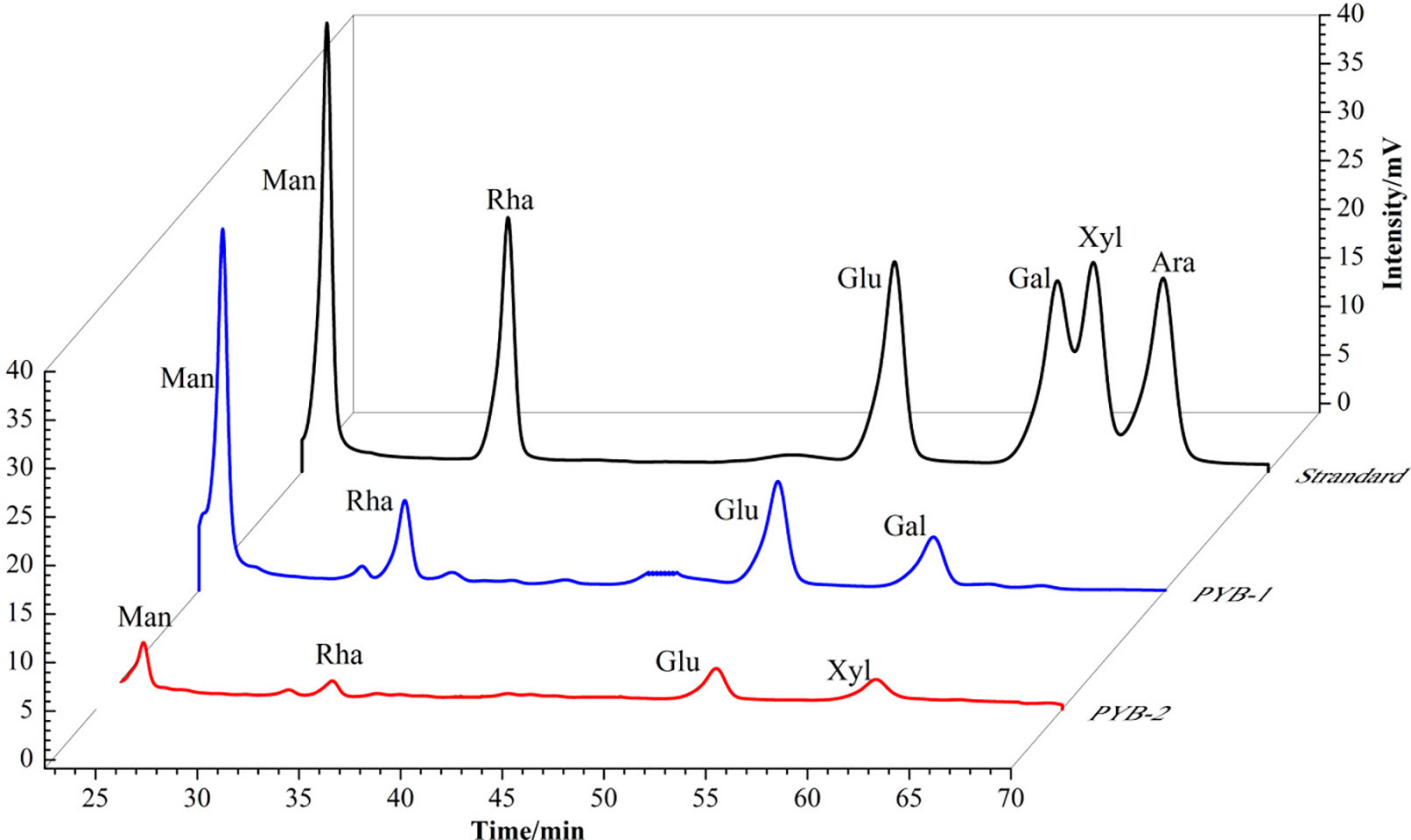
High‐performance liquid chromatograms comparing monosaccharide standards at different concentrations (0.2, 0.4, 0.6, 0.8, and 1.0 mg/mL) with polysaccharides from *yam bulbils* (PYB‐1 and PYB‐2).

With respect to retention time, the four peaks in HPLC chromatograms of PYB‐1 and PYB‐2 matched peaks in the standard monosaccharide sample (Figure 2). The PYB‐1 fraction comprised Man, Rha, Glu, and Gal at a molar ratio of 2.19:0.88:1.45:0.58. Likewise, PYB‐2 consisted of Man, Rha, Glu, and Xyl, but at a molar ratio of 0.61:0.34:0.39:0.16. Thus, Man and Glu were the major monosaccharides in PYB‐1 and PYB‐2.

**FIGURE 3:**
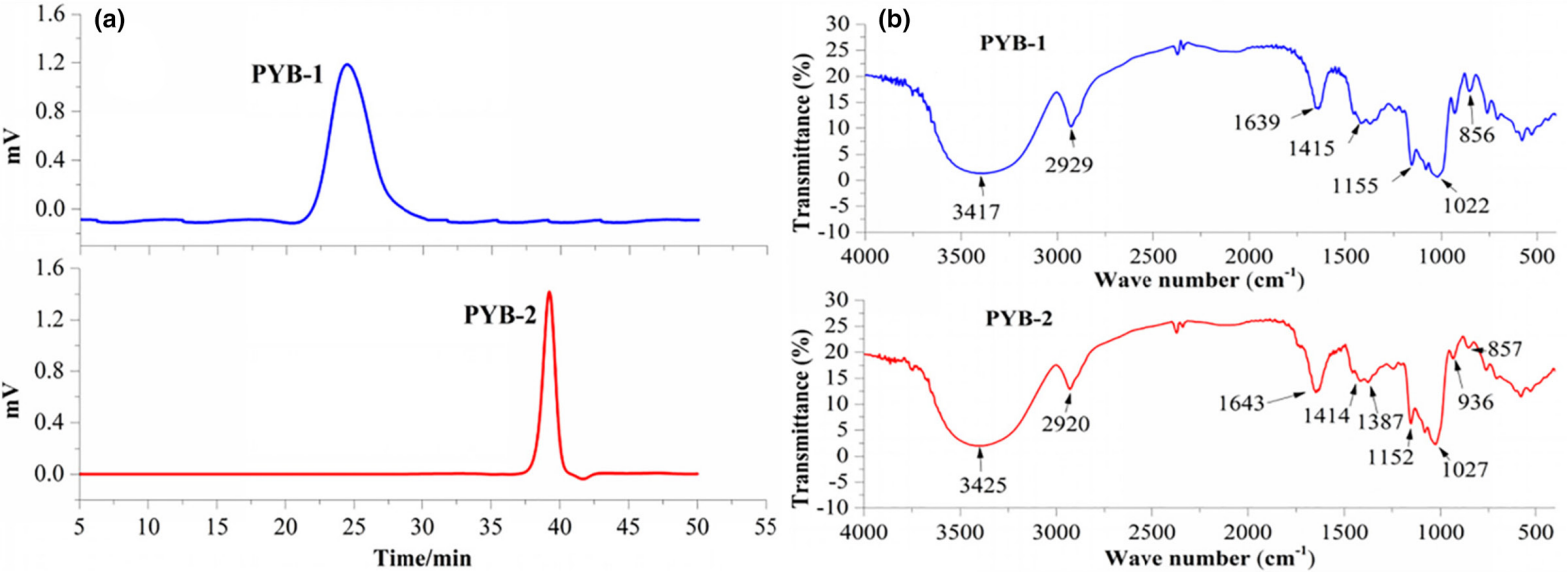
Structural characteristics of polysaccharides from *yam bulbils* (PYB‐1/2), determined with gel permeation chromatography (a) and Fourier‐transform infrared (b).

The GPC profile (Figure 3a) revealed that PYB‐1 and PYB‐2 had average Mw of 145,423 and 11,318 Da. Hydroxyl (3417 cm^−1^), methyl (2929 cm^−1^), and α‐/β‐configurations (856 and 1022 cm^−1^) were observed (Figure 3b) (Yang et al., 2014; Zhou et al., 2022), confirming the presence of α‐ and β‐sugar residues in PYB‐1. Similarly, for PYB‐2, absorption bands for hydroxyl (3425 cm^−1^), methyl (2920 cm^−1^), uronic acid (1414 cm^−1^), and α‐/β‐configurations (857, 1027, and 936 cm^−1^) were visible.

### 3.3 Morphological analysis of PYB

**FIGURE 4:**
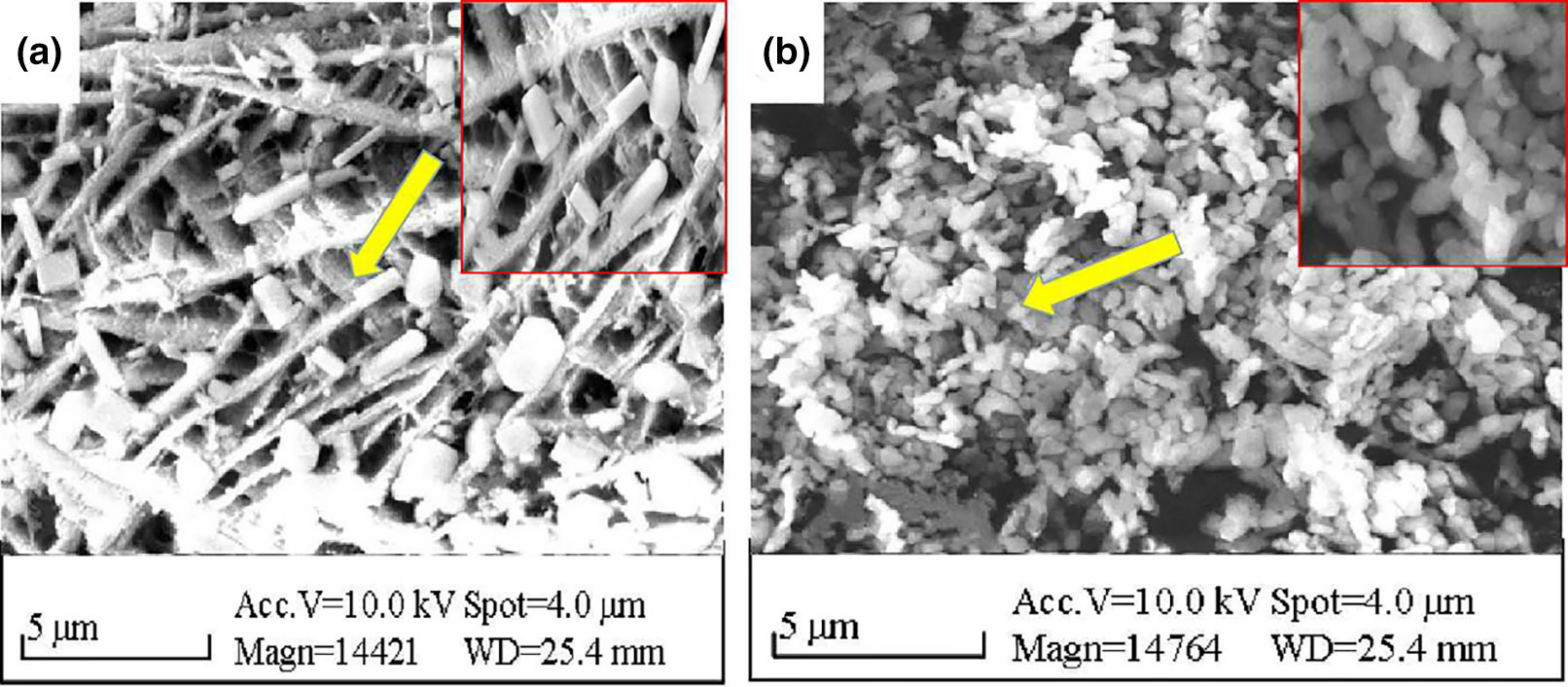
Morphological properties of PYB‐1 (a) and PYB‐2 (b) via scanning electron microscopy.

Their complex forms result in polysaccharides possessing distinct morphological properties (Ji et al., 2021). Here, SEM images revealed that PYB‐1 (Figure 4a) was loose and porous with irregular rod shapes. In contrast, PYB‐2 (Figure 4b) exhibited a smooth surface and spheroidal structure, with strong interactions between individual polysaccharides.

### 3.4 Antioxidant activities of PYB in vitro

The radical theory posits that free radical accumulation is the direct cause of fatigue (Miao et al., 2018). Thus, biologically active substances with antifatigue activity should also exhibit antioxidant effects with free radical scavenging activity. Therefore, antioxidant activity in PYB was measured.

#### 3.4.1 DPPH scavenging ability

**FIGURE 5:**
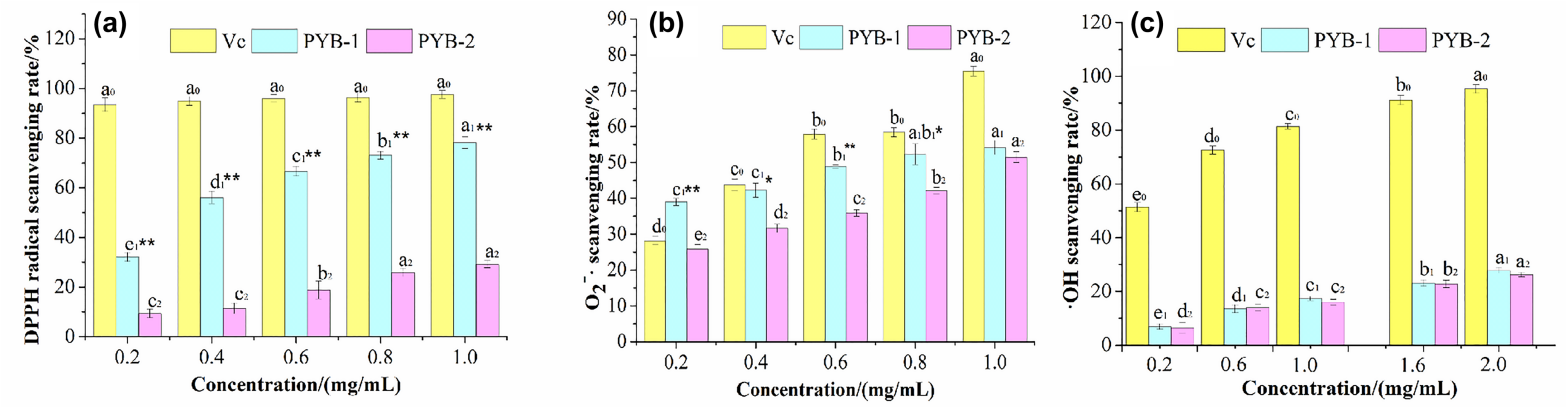
Scavenging abilities of polysaccharides from *yam bulbils* (PYB‐1/2) on DPPH free radical, superoxide anion free radical, and hydroxyl free radical. Different letters indicate significant differences between groups (*p* < .05). Significant differences between PYB‐1 and PYB‐2 (*p < .05, **p < .01).

Polysaccharides contain many hydroxyl groups that act as hydrogen electron donors, reacting with and stabilizing DPPH. The DPPH scavenging rates of PYB‐1 and PYB‐2 were both dose dependent (Figure 5a). At 1.0 mg/mL, PYB‐1 and PYB‐2 scavenging rates were 78.22 ± 2.29% and 29.14 ± 1.51%, respectively, clearly indicating that PYB‐1 had higher DPPH scavenging capacity than PYB‐2 (Figure 5a).

#### 3.4.2 Superoxide anion scavenging ability

As with DPPH, the $O_{2}^{-\cdot }$ scavenging ability of PYB‐1 and PYB‐2 was dose dependent (Figure 5b). At a concentration of 1.0 mg/mL, the scavenging rate of PYB‐1 and PYB‐2 scavenging rates was 54.12 ± 1.97% and 51.51 ± 1.49%, respectively, showing the superior antioxidant capacity of PYB‐1.

#### 3.4.3 Hydroxyl scavenging ability

Hydroxyl (·OH) is a highly reactive free radical that easily penetrates cell membranes, causing damage and even death. Scavenging ·OH is, thus, a particularly valuable characteristic (Qiao et al., 2022). It found that the ·OH scavenging abilities of PYB‐1 and PYB‐2 exhibited a dose‐dependent relationship (Figure 5c), but their rates did not differ (*p* > .05).

The antioxidant test results revealed that PYB‐1 had superior scavenging capacity for DPPH and $O_{2}^{-\cdot }$ than PYB‐2 while equaling the latter for ·OH. Both PYB fractions had significantly lower scavenging capacity than Vc for all three free radicals.

### 3.5 Antifatigue activity of PYB

#### 3.5.1 Effects of PYB‐1 and PYB‐2 on exhaustive swimming time, body weight, and organ indices in mice

The effects of PYB‐1 and PYB‐2 on exhaustive swimming time, body weight, and organ indices in mice are shown in Table 1. Growth rates and organ indices did not differ among the eight groups. Therefore, mouse metabolism and immunity were not significantly altered by oral administration of PYB‐1 and PYB‐2 at different concentrations for 4 weeks. All mice remained healthy, with no anomalies in behavior or appearance throughout the experimental period, indicating the lack of side effects.

**TABLE 1:** Effect of polysaccharides from *yam bulbils* on body weight and organ indices in mice.

Groups	Growth rate (%)	Organ indices			Exhaustive swimming time (min)
		Liver (%)	Spleen (%)	Lung (%)	
CG	32.02 ± 1.47^a^	5.34 ± 0.60^a^	0.30 ± 0.06^a^	0.59 ± 0.06^a^	57.48 ± 6.63^f^
PYB‐1 LG	32.25 ± 1.79^a^	5.38 ± 0.75^a^	0.27 ± 0.07^a^	0.55 ± 0.03^a^	94.76 ± 8.09^d,##^
PYB‐1 MG	28.67 ± 1.59^a^	5.25 ± 0.10^a^	0.28 ± 0.04^a^	0.58 ± 0.03^a^	120.39 ± 7.44^ab,##^
PYB‐1 HG	28.42 ± 1.45^a^	5.26 ± 0.52^a^	0.29 ± 0.04^a^	0.56 ± 0.03^a^	124.32 ± 6.46^a,#^
PYB‐2 LG	30.37 ± 1.48^a^	5.36 ± 0.89^a^	0.28 ± 0.03^a^	0.57 ± 0.03^a^	80.12 ± 5.14^e^
PYB‐2 MG	29.87 ± 1.88^a^	5.28 ± 0.23^a^	0.27 ± 0.02^a^	0.58 ± 0.02^a^	107.89 ± 5.18^c^
PYB‐2 HG	28.78 ± 1.18^a^	5.28 ± 0.16^a^	0.28 ± 0.04^a^	0.59 ± 0.03^a^	112.67 ± 4.15^bc^
PG	30.89 ± 1.96^a^	5.29 ± 0.36^a^	0.28 ± 0.03^a^.	0.57 ± 0.02^a^	126.16 ± 6.75^a^

*Note*: Different letters indicate significant differences between groups (ANOVA, *p* < .05). Control, CG; low‐dose PYB, LG; medium‐dose PYB, MG; high‐dose PYB, HG; positive control, PG.

Abbreviations: CG, Control; HG, high‐dose PYB; LG, low‐dose PYB; MG, medium‐dose PYB; PG, positive control.

Significant differences between PYB‐1 and PYB‐2 (^#^ *p* < .05, ^##^ *p* < .01).

The antifatigue activity of PYB‐1 and PYB‐2 was investigated using EST. Supplementation with PYB‐1, PYB‐2, and positive control all increased swimming time significantly above CG levels (*p* < .05; Table 1). Therefore, increasing polysaccharide doses could prolong exhaustive swimming time in mice. Additionally, both MG and HG mice had greater endurance than LG mice (*p* < .05), whereas MG, HG, and PC mice did not differ when supplemented with PYB‐1 (*p* > .05). These two kinds of polysaccharides significantly increased the swimming time at different doses (*p* < .05), in which the effects of polysaccharides with higher molecular weights were more significant (*p* < .05). These results are similar to those obtained by Liu et al. (2015), suggesting that different PYB doses elevate endurance during exercise. Taken together, the data suggest that PYB exerts antifatigue effects and PYB‐1 had an intuitive effect in relieving physical fatigue.

#### 3.5.2 Effects of PYB on biochemical parameters after swimming

To further characterize the antifatigue effects of PYB‐1 and PYB‐2, the biochemical indices were measured. During intensive exercise, sugar or fat catabolism is insufficient for energy needs, leading to protein and amino acid metabolism, with BUN as a by‐product (Xu et al., 2017; Yang et al., 2016; Zhu, Yang, et al., 2021). Thus, BUN is positively correlated with exercise endurance (Yan et al., 2020) and indicates the degree of fatigue (Ara et al., 2018).

**FIGURE 6:**
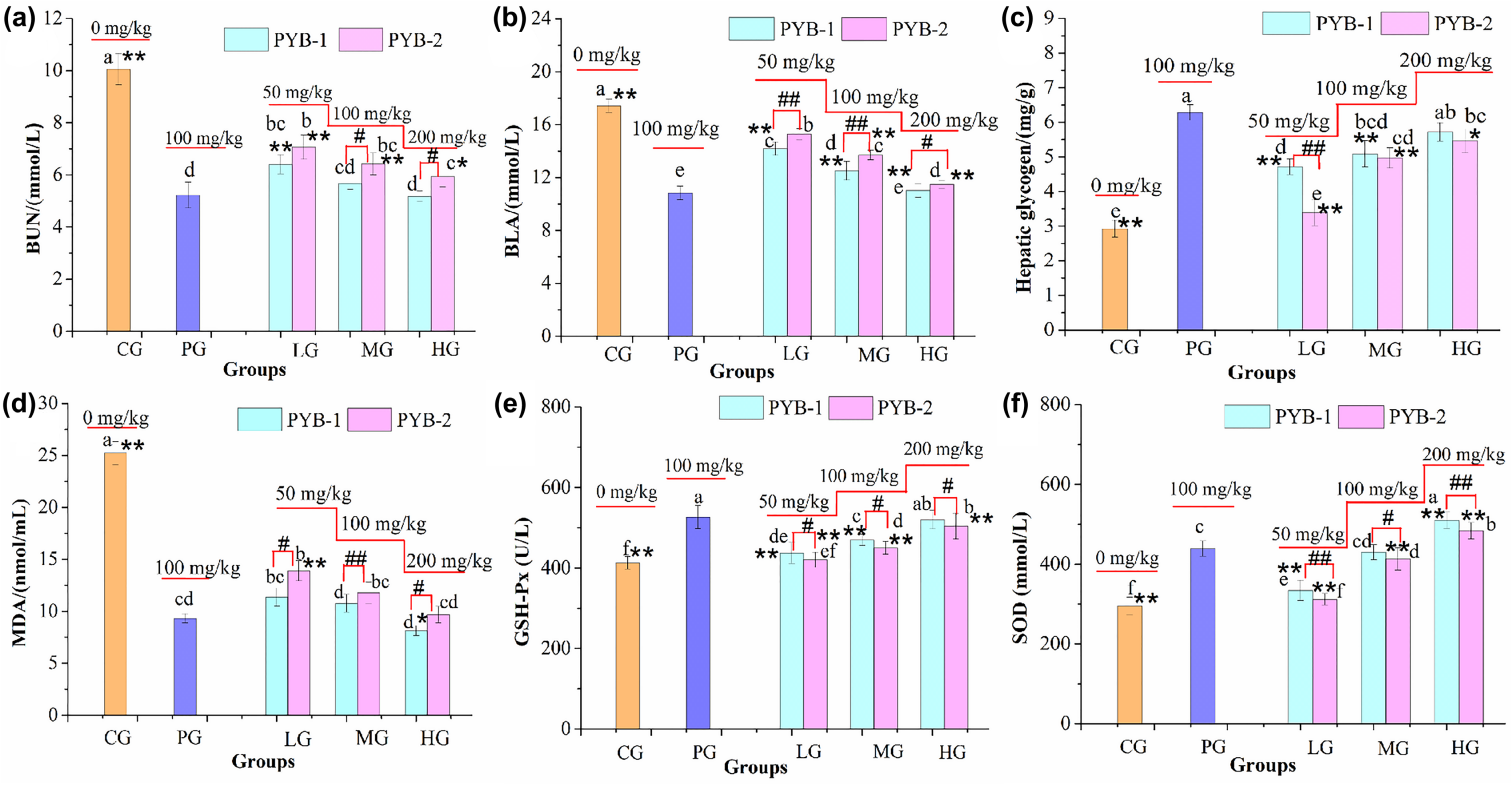
Effect of PYB‐1 and PYB‐2 on internal biochemical indices. Significant differences between PYB‐1 and PYB‐2 (^#^ *p* < .05, ^##^ *p* < .01). Compared with PG, the significant differences among other groups (*p < .05, **p < .01).

Here, in Figure 6a, it was found that all treatment groups had significantly lower BUN concentrations than CG (*p* < .05). Thus, PYB‐1 and PYB‐2 decreased BUN levels after exhaustive swimming in a dose‐dependent manner, indicative of antifatigue effects. Overall, BUN levels of treatment groups and PG differed significantly (*p* < .05, *p* < .01), except in HG and PYB‐1 MG (*p* > .05). Significant changes in BUN levels were found in mice treated with PYB‐1 (100 mg/Kg, 200 mg/Kg) compared with those in PYB‐2.

The end‐product of anaerobic glycolysis (Yang et al., 2022), BLA accumulates during high‐intensity physical labor and inhibits muscle contraction to induce fatigue (Zhao et al., 2018). Thus, BLA is a crucial index of postexercise fatigue (Surhio et al., 2017).

After EST, CG had significantly higher BLA levels (*p* < .05) than treatment groups (Figure 6b). The BLA level in the PG was 10.82 ± 0.52 mmol/L. Compared with the PG, the BLA level of mice in the other treated groups increased significantly (*p* < .05, *p* < .01); however, no significant changes were found in PYB‐1 HG with 11.02 ± 0.52 mmol/L (*p* > .05). Compared with PYB‐1, the BLA level of mice in PYB‐2 LG, MG, and HG was 15.29 ± 0.43, 13.70 ± 0.37, and 11.58 ± 0.29 mmol/L, respectively, which were found significantly different between those in the PYB‐1 groups (*p* < .05, *p* < .01). These findings showed that PYB inhibited post‐EST increases in BLA levels, suggesting that the two extracted polysaccharides can prevent lactic acidosis.

Energy consumption and deficiency are the main causes of physical fatigue and decreased endurance during exercise (Li et al., 2017). Energy for heavy exercise comes from circulating glucose released by the liver after depleting muscle glycogen. Thus, increasing hepatic glycogen storage is important for increasing endurance and exercise capacity (Martins et al., 2018). As shown in Figure 6c, the hepatic glycogen level in CG was 2.92 ± 0.24 mg/g. Compared with CG, the hepatic glycogen levels of mice in the treatment groups increased significantly (*p* < .05), especially PG, with 6.28 ± 0.22 mg/g. Furthermore, hepatic glycogen content was positively correlated with PYB dose. Compared with PG, the hepatic glycogen levels of mice were found significantly different in the PYB‐2 groups, PYB‐1 LG and PYB‐1 MG (*p* < .05, *p* < .01). However, no significant changes in hepatic glycogen levels were found compared with PG and PYB‐1HG (*p* > .05). Meanwhile, there were no significant differences in hepatic glycogen levels between PYB‐1 groups (100–200 mg/kg) (*p* > .05) and those in the PYB‐2, except for PYB‐1 LG and PYB‐2 LG (50 mg/kg) (*p* < .01). These results demonstrated that PYB‐1 and PYB‐2 could increase hepatic glycogen storage helping enhance endurance.

Intense exercise disrupts the equilibrium between the antioxidant and oxidant systems, resulting in the release of reactive oxygen species (ROS). Excess ROS breaks down polyunsaturated fatty acids to cause lipid peroxidation, producing MDA as a by‐product (Qi et al., 2014; Yang et al., 2014, 2015; Zhou et al., 2022). Thus, fatigue increases MDA levels (Yang et al., 2022; Zhao et al., 2017).

Figure 6d shows that MDA contents in all treatment groups decreased significantly (*p* < .05), of which PYB‐1HG was the most significant (8.15 ± 0.48 nmol/L, 67.7%). Compared with PG, the MDA levels of mice showed significant differences in the PYB‐2 LG and PYB‐1 HG (*p* < .05, *p* < .01). And the significant changes in MDA levels of mice were found by comparing PYB‐1 groups with those of PYB‐2 groups. Therefore, the antifatigue effects of PYB‐1 and PYB‐2 may largely stem from lowering lipid peroxidation and preventing breakdown of corpuscular membranes (Ni et al., 2013; Yang et al., 2015).

Intense exercise consumes a large amount of oxygen, accelerating the production of free radicals and ROS (Qiao et al., 2022) to cause oxidative stress. Oxidative stress plays a significant role in physical fatigue. Therefore, the antioxidants SOD and GSH‐Px, which are well documented to protect cells against oxidative stress through decreasing ROS production and eliminating harmful metabolic products, were evaluated (Li & Lu, 2022). Under continuous PYB‐1/2 administration, SOD (Figure 6e) and GSH‐Px (Figure 6f) activities significantly increased (*p* < .05) from CG levels, while MDA levels decreased (*p* < .05). It also was observed that SOD and GSH‐Px activities did not differ from PG levels after treatment with PYB‐1MG, confirming that the PYB‐1 MG has the same effect as 100 mg/mL of LPRC. And the significant changes in SOD and GSH‐Px activities of mice were found by comparing PYB‐1 groups with those of PYB‐2 groups (*p* < .05, *p* < .01).

Taken together, the experimental data revealed that PYB‐1 and PYB‐2 exerted their antifatigue effects in mice.

## 4 DISCUSSION

In this study, the structures of two polysaccharides (PYB‐1 and PYB‐2) obtained from Chinese *yam bulbils* by UAE were analyzed. They were composed of Man, Rha, Glc, Gal, and Xyl in different proportions and had molecular weights of 1.45 × 10^5^ and 1.13 × 10^4^ Da. PYB‐1 and PYB‐2 all had antifatigue activity; however, PYB‐1 exhibited better antifatigue and antioxidant activities than PYB‐2.

Fatigue is defined as trouble in sustaining or initiating voluntary activities, resulting from hard physical or mental work and severe stress (Nakagawasai et al., 2021), which becomes common in people's lives. However, antifatigue drugs often have several negative effects. Thus, it is significant to find new antifatigue agents with definite efficacy and fewer side effects. Fatigue is a complex and comprehensive physiological phenomenon, which is related to excessive consumption of energy and substances metabolite accumulation. When the human body exercises for a long time or violently, the acceleration of metabolism and muscle contraction leads to the massive accumulation of metabolic wastes, such as BLA and BUN, in cells and destruction of the internal environment of tissues, thereby resulting in fatigue. Meanwhile, exercise promotes glycogen consumption and accelerates the decomposition of liver glycogen, which is converted into glucose to supply energy, resulting in decreasing the level of liver glycogen. This research showed that PYB can effectively extend forced swimming time and increase fatigue in mice liver glycogen reserves and decrease the levels of BUN and BLA. These findings indicate that PYB supply energy substances to relieve fatigue.

Furthermore, during exercise, the excessive reactive oxygen species (ROS) can be produced, and excessive free radicals in the body cause cell metabolism disorder and lipid peroxidation, leading to fatigue (Peng et al., 2021). MDA, a toxic product generated from lipid peroxidation, is typically used as an indicator to evaluate oxidative damage in the body. SOD and GSH‐Px are important antioxidant enzymes that can reduce the oxidative damage induced by free radical. In this study, the results showed that PYB have antioxidant activity via scavenging free radical in vitro, especially PYB‐1, increase SOD and GSH‐Px activities, and decrease the level of MDA in fatigue mice.

Some studies have shown that the biological activity of polysaccharides is affected by their structure. Molecular weight is one of the basic indicators of polysaccharides and an important factor affecting their biological activity (Liu et al., 2015; Liu, Gou, et al., 2022; Liu, Yang, et al., 2022). Cai et al. (2021) found that the polysaccharides with higher Mw had better antifatigue effect than the lower Mw. In the present study, similar results were obtained in antifatigue experiment. Compared with PYB‐2, PYB‐1 with higher molecular weight (PYB‐1 is about 12.8 times that of PYB‐2) showed more effective antifatigue and antioxidant activities. Monosaccharide composition can indicate the relationship between polysaccharide structure and its function. Studies show that uronic acid can affect the bioactivity of polysaccharides (Li et al., 2021). In this study, PYB‐1 and PYB‐2 all had uronic acid (1414 cm^−1^), but the PYB‐1 had better antifatigue and antioxidant activities than PYB‐2. These results illustrated the hypothesis that the influence of Mw on the antifatigue and antioxidant activities of PYB may be better than that of monosaccharide composition.

## 5 CONCLUSION

In conclusion, two polysaccharides (PYB‐1 and PYB‐2) were isolated from Chinese *yam bulbils* and provided evidence of their antifatigue effects, as well as demonstrating that the mechanism of antifatigue and antioxidant activities may be related to their molecular weight. In addition, it was also observed that administering the same concentrations (100 mg/mL) of PYB‐1 and LPRC resulted in similar swimming times and biological parameters (*p* > .05), indicating that the two compounds have equally strong antifatigue effects. PYB‐1 showed significant antifatigue effects by rising hepatic glycogen content and antioxidant enzyme (SOD, GSH‐Px) activity, declining the levels of BUN, BLA, and MDA, suggesting that PYB‐1 can be developed as a novel agent to alleviate fatigue.

## AUTHOR CONTRIBUTIONS

**Hai‐Xu Zhou:** Funding acquisition (supporting); methodology (equal); project administration (equal); writing – review and editing (equal). **Xiao Zhang:** Methodology (equal); writing – original draft (equal). **Ren‐gui Huang:** Methodology (equal); writing – review and editing (equal). **Tong‐chao Su:** Formal analysis (equal); funding acquisition (supporting); software (equal).

## CONFLICT OF INTEREST STATEMENT

The authors declare no conflicts of interest.

## ETHICS STATEMENT

The study does not involve any human testing.

## INSTITUTIONAL REVIEW BOARD STATEMENT

The study was approved by the Committee of Experimental Animal Welfare and Ethics of Henan Institute of Science and Technology of College of Animal Science and Veterinary Medicine (approved on 16 March, 2021; approval number PZASVM2103160016).

## Data Availability

The data that support the findings of this study are available from the corresponding author upon reasonable request.
